# Crystal structure and electrochemical properties of [Ni(bztmpen)(CH_3_CN)](BF_4_)_2_ {bztmpen is *N*-benzyl-*N*,*N*′,*N*′-tris­[(6-methyl­pyridin-2-yl)meth­yl]ethane-1,2-di­amine}

**DOI:** 10.1107/S2056989017006764

**Published:** 2017-05-09

**Authors:** Lin Chen, Gan Ren, Yakun Guo, Ge Sang

**Affiliations:** a Science and Technology on Surface Physics and Chemistry Laboratory, Jiangyou 621908, People’s Republic of China; b Institute of Materials, China Academy of Engineering Physics, Jiangyou 621908, People’s Republic of China

**Keywords:** crystal structure, nickel, poly-pyridine-di­amine, electro-catalyst

## Abstract

The mononuclear nickel title complex (acetonitrile-κ*N*){*N*-benzyl-*N*,*N*′,*N*′-tris­[(6-methyl­pyridin-2-yl)meth­yl]ethane-1,2-di­amine}­nickel(II) bis­(tetra­fluor­ido­borate), [Ni(C_30_H_35_N_5_)(CH_3_CN)](BF_4_)_2_, was prepared from the reaction of Ni(BF_4_)_2_·6H_2_O with *N*-benzyl-*N*,*N*′,*N*′-tris­[(6-methyl­pyridin-2-yl)meth­yl]ethane-1,2-di­amine (*bztmpen*) in aceto­nitrile at room temperature. With an open site occupied by the aceto­nitrile mol­ecule, the nickel(II) atom is chelated by five N-atom sites from the ligand and one N atom from the ligand, showing an overall octa­hedral coordination environment. Compared with analogues where the 6–methyl substituent is absent, the bond length around the Ni^2+^ cation are evidently longer. Upon reductive dissociation of the acetro­nitrile mol­ecule, the title complex has an open site for a catalytic reaction. The title complex has two redox couples at −1.50 and −1.80 V (*versus F*_c_^+/0^) based on nickel. The F atoms of the two BF_4_^−^ counter-anions are split into two groups and the occupancy ratios refined to 0.611 (18):0.389 (18) and 0.71 (2):0.29 (2).

## Abstract 2

The structure and electrochemical properties of a nickel tri­pyridine–di­amine complex are reported. The complex has two redox couples at −1.50 and −1.80 V (*versus F*_c_^+/0^) based on nickel.

## Chemical context   

Nickel complexes with polypyridine–amine ligands are of great inter­est in catalytic reactions. For example, nickel complexes containing N5-penta­dentate ligands with different amine-to-pyridine ratios have been studied for electrochemical H_2_ production in water at pH = 7 and the complex with a di­amine–tri­pyridine ligand displays a TON (turn-over number) of up to 308000 over 60 h electrolysis at −1.25 V *vs* the standard hydrogen electrode (SHE), with a Faradaic efficiency of 91% (Zhang *et al.*, 2014 ▸). The nickel-based complex Ni–PY_5_ {PY_5_ = 2,6-bis­[1,1-bis­(2-pyrid­yl)eth­yl]pyridine} has been found to act as an electro-catalyst for oxidizing water to di­oxy­gen in aqueous phosphate buffer solutions (Wang *et al.*, 2016 ▸). The rate of water oxidation catalyzed by the Ni–PY_5_ complex is enhanced remarkably by the proton-acceptor base HPO_4_^2−^, with a rate constant of 1820 M^−1^ s^−1^. A stable configuration is important for the stability of a catalyst. In the title complex, the Ni^2+^ cation is chelated by five N-atom sites, so the configuration is stable. With the reductive dissociation of aceto­nitrile, the title complex would give an open site for a catalytic reaction. Herein, we describe the crystal structure and electrochemical properties of the title complex.
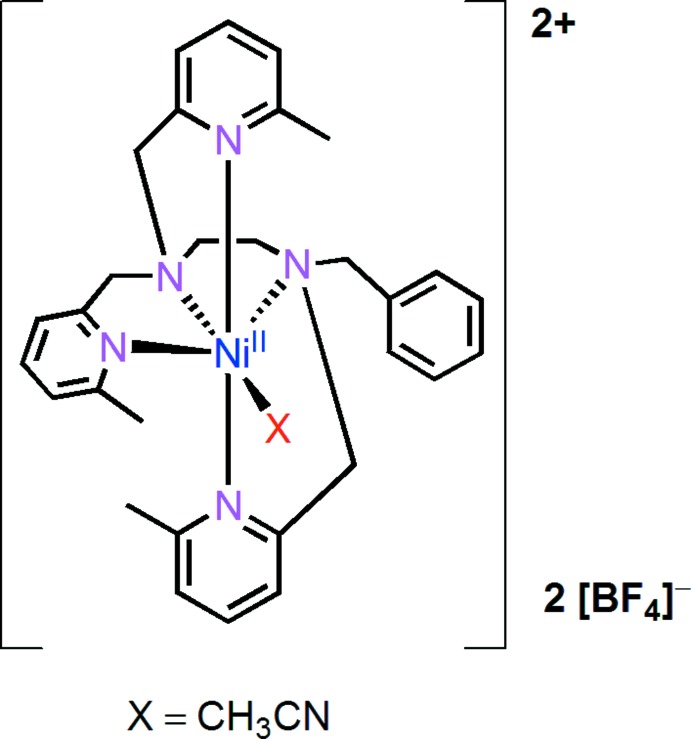


## Structural commentary   

**Figure 1:**
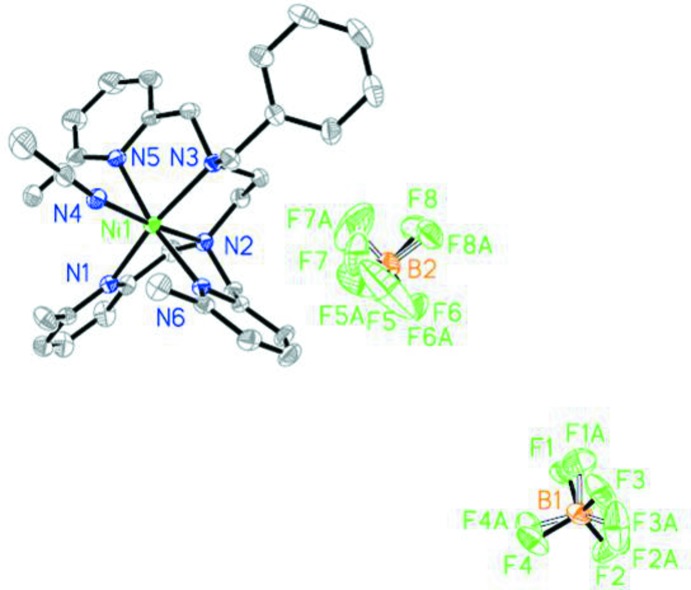
The structures of the molecular components in the title compound, showing 50% probability displacement ellipsoids. H atoms have been omitted for clarity.

In the title complex (Fig. 1 ▸), the coordination sphere of the nickel(II) atom adopts a normal octa­hedral geometry. The Ni^2+^ cation lies almost in the equatorial plane. One pyridine nitro­gen atom (N1) and two amino nitro­gen atoms (N2, N3) as well as the nitrogen atom of an acetonitrile ligand (N4) form the equatorial plane. The latter can easily be dissociated from nickel. The axial positions are occupied by two pyridine nitro­gen atoms (N5, N6). The Ni—N bond lengths for the two axial pyridine–nitro­gen atoms [Ni—N5 = 2.209 (3) and Ni—N6 = 2.187 (3) Å] are significantly longer than that for the other four nitro­gen atoms [Ni—N1 = 2.151 (3), Ni—N2 = 2.082 (3), Ni—N3 = 2.188 (2), Ni—N4 = 2.061 (3) Å]. The presence of the 6-methyl substit­uent hinders the approach of the pyridine group to the Ni^2+^ core. A a result of the steric hindrance from the methyl substituent, the three atoms N5, Ni1 and N6 are not completely linear in the axial direction, with a contact angle of 170.89 (9)°. Two intra­molecular C—H⋯N contacts occur (Table 1 ▸).

## Electrochemical commentary   

**Figure 2:**
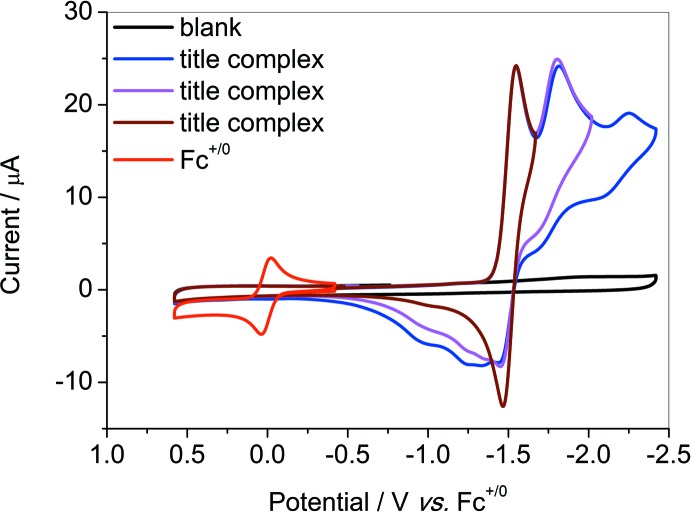
Cyclic voltammograms of the title complex (1 m*M*) with a varied scan range under Ar in CH_3_CN with 0.1 *M ^n^*Bu_4_NBF_4_ as the supporting electrolyte.

**Figure 3:**
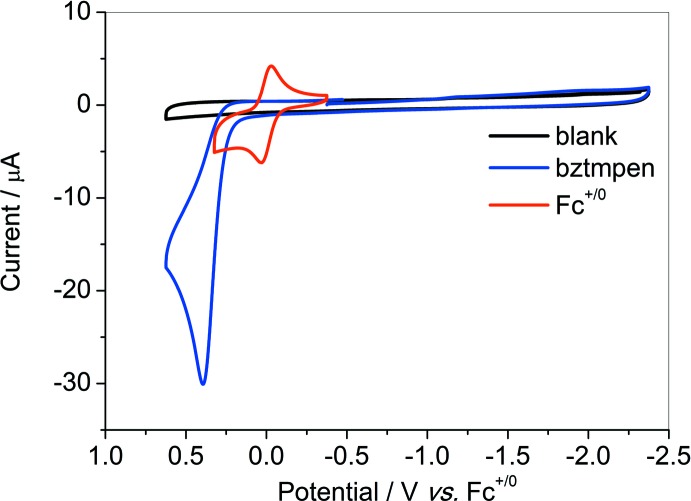
Cyclic voltammograms of ligand *bztmpen* (1 m*M*) under Ar in CH_3_CN with 0.1 *M ^n^*Bu_4_NBF_4_ as the supporting electrolyte.

Generally, the reduction of a metal complex is accompanied by the dissociation of the ligand, or the weakest ligand if more than one ligand is present, which could induce the appearance of an open site for a catalytic reaction (Knoll *et al.*, 2014 ▸; Johnson *et al.*, 2016 ▸). The introduction of *o*-methyl in the title complex is in favor of the dissociation of aceto­nitrile. On the cathodic scan under Ar, the title complex features one reversible couple at −1.50 V and one half-reversible couple at −1.80 V (*vs F*_c_^+/0^) based on nickel, assigned to Ni^II/I^ and Ni^I/0^ respectively (Fig. 2 ▸). The third couple at −2.15 V could be assigned to the reduction of pyridine. The free ligand *bztmpen* itself is electrochemically silent in the potential range (Fig. 3 ▸). The coordination with nickel leads to a positive shift of the reduction on pyridine. The good reversibility of the couple indicates a negligible change in the configuration of the title complex after one electron reduction. The second reduction might result in a change of the configuration. Analogues in the absence of *o*-methyl show only one redox couple more negative than −1.50 V (*vs F_c_^+/0^*; Zhang *et al.*, 2014 ▸). The positive shift of the first redox couple for the title complex results from the weaker electron-donating ability of the pyridine ligands, which are farther from the nickel core. The electrochemical properties of these analogues are consistent with the differences shown in the structure.

## Supra­molecular features   

**Figure 4:**
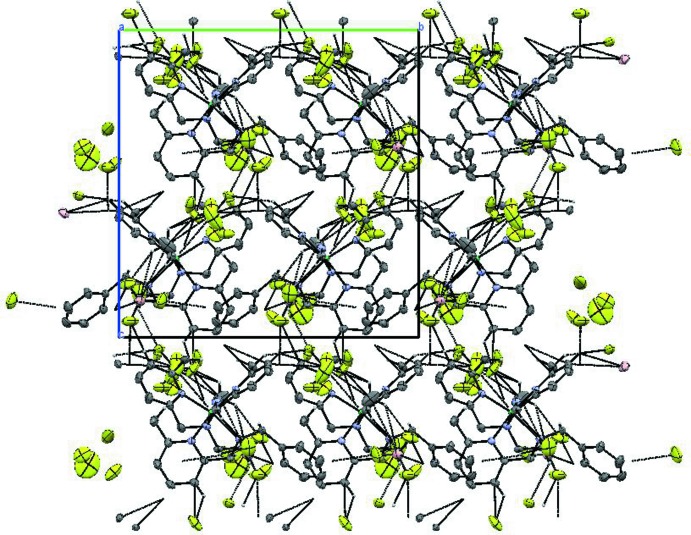
Packing plot of the mol­ecular components in the title compound viewed down the *a* axis. C—H⋯F weak bonds are shown as dotted lines.

In the title crystal, no classical hydrogen bonds have been found. Weak C—H⋯F contacts (Table 1 ▸) link the components into a three-dimensional network. The crytal paacking is illustrated in Fig. 4 ▸.

## Database survey   

There are three published nickel complexes with poly-pyridine groups (Shi *et al.*, 2015 ▸; Zhang *et al.*, 2014 ▸; Wang *et al.*, 2016 ▸), but to the best of our knowledge, the title compound has not been reported previously. The nickel complex with *N*,*N*,*N*′,*N*′-tetra­(2-pyridyl­meth­yl)ethyl­enedi­amine (*tpen*) adopts a normal octa­hedral geometry (Shi *et al.*, 2015 ▸). In the Ni^2+^(tpen) complex, the Ni—N1, Ni—N2, Ni—N3, Ni—N4, Ni—N5 and Ni—N6 bonds [2.106 (3), 2.099 (3), 2.114 (3), 2.086 (3), 2.094 (3) and 2.120 (2) Å, respectively] are shorter than the corresponding bond lengths in the title complex. Among the earliest reports, the nickel complex with *N*-benzyl-*N*,*N*′,*N*′-tris­(2-pyridyl­meth­yl)ethyl­enedi­amine (*bztpen*) ligand is most similar to the title complex (Zhang *et al.*, 2014 ▸). Under reductive conditions, Ni^2+^(*bztpen*) displays a high activity on electro-catalytic water reduction. The title complex possesses a higher steric hindrance than Ni^2+^(bztpen), which affects evidently the bond lengths, especially in the axial direction. The bonds lengths in the title complex [Ni—N5 = 2.209 (3), Ni—N6 = 2.187 (3) Å] are longer than those in Ni^2+^(*bztpen*) [Ni—N5 = 2.149 (3), Ni—N6 = 2.096 (3) Å]. The nickel complex with a PY_5_ ligand {PY_5_ = 2,6-bis­[1,1-bis­(2-pyrid­yl)eth­yl]pyridine} displays a similar configuration to the title complex, but the labile ligand is at the axial site (Wang *et al.*, 2016 ▸). Ni^2+^(PY_5_) has been found to act as an electro-catalyst for oxidizing water to di­oxy­gen in an aqueous phosphate buffer solution.

## Synthesis and crystallization   

The tri­pyridine-di­amine ligand *N*-benzyl-*N*,*N*′,*N*′-tris­[(6-methyl­pyridin-2-yl)meth­yl]ethane-1,2-di­amine (*bztmpen*) was prepared according to literature procedures (Zhang *et al.*, 2013 ▸), ^1^H NMR (CDCl_3_, 600 MHz): *δ* 7.44 (*m*, 4H), 7.25 (*m*, 6H), 6.96 (*m*, 4H), 3.74 (*s*, 6H), 3.58 (*s*, 2H), 2.75 (*d*, 4H), 2.49 (*s*, 9H). ESI–MS: calculated for [*M* + H]^+^: *m*/*z* 466.63; found: 466.27.

Preparation of [Ni(*bztmpen*)(CH_3_CN)](BF_4_)_2_. Compound Ni(BF_4_)_2_·6H_2_O (0.16 g, 0.5 mmol) was added to an aceto­nitrile solution (5 mL) of *bztmpen* (0.2 g, 0.5 mmol). The mixture was stirred at room temperature for 6 h. The purple solution was then transferred to tubes, which were placed in a flask containing ether. Block-shaped blue crystals were obtained in a yield of 85% (0.25 g). Analysis calculated for C_32_H_38_B_2_F_8_N_6_Ni (%): C, 50.01; H, 5.18; N, 11.37; found: 50.01; H, 5.19; N, 11.36; MS (TOF–ES): *m*/*z* = 282.6341 {[*M*−2BF_4_^−^]/2}^+^, 599.3015 [*M* – 2BF_4_^−^ + Cl^−^]^+^.

## Refinement   

Crystal data, data collection and structure refinement details are summarized in Table 2 ▸. The F atoms of the two BF_4_^−^ counter-anions were split into two groups and the occupancies refined to 0.611 (18)/0.389 (18) and 0.71 (2)/0.29 (2). The hydrogen atoms were refined in a riding mode with C—H = 0.93–0.97 Å and *U*_iso_(H) = 1.2*U*_eq_(C).

## Supplementary Material

Crystal structure: contains datablock(s) I. DOI: 10.1107/S2056989017006764/vn2128sup1.cif

Structure factors: contains datablock(s) I. DOI: 10.1107/S2056989017006764/vn2128Isup4.hkl

CCDC reference: 1548052

Additional supporting information:  crystallographic information; 3D view; checkCIF report

## Figures and Tables

**Table 1:** Hydrogen-bond geometry (Å, °)

*D*—H⋯*A*	*D*—H	H⋯*A*	*D*⋯*A*	*D*—H⋯*A*
C1—H1*C*⋯N4	0.96	2.94	3.251 (6)	100
C3—H3*A*⋯F8^i^	0.93	2.57	3.457 (14)	159
C8—H8*B*⋯F7	0.97	2.57	3.371 (13)	140
C8—H8*B*⋯F5*A*	0.97	2.42	3.36 (4)	163
C9—H9*B*⋯F4^ii^	0.97	2.61	3.291 (16)	128
C10—H10*A*⋯N6	0.97	2.67	3.273 (5)	1213
C12—H12*A*⋯F8*A* ^iii^	0.93	2.53	3.35 (2)	146
C17—H17*B*⋯N1	0.96	2.64	3.071 (7)	108
C21—H21*A*⋯F3^ii^	0.93	2.62	3.122 (10)	114
C23—H23*B*⋯F6*A* ^iii^	0.97	2.33	3.241 (16)	157
C24—H24*A*⋯F7	0.97	2.53	3.443 (15)	156
C24—H24*B*⋯F3^iv^	0.97	2.32	3.179 (13)	148
C24—H24*B*⋯F1*A* ^iv^	0.97	2.54	3.43 (3)	153
C28—H28*A*⋯F4^iii^	0.93	2.59	3.345 (19)	138
C30—H30*A*⋯F4^iii^	0.96	2.60	3.390 (17)	140
C30—H30*A*⋯F4*A* ^iii^	0.96	2.50	3.26 (3)	136
C30—H30*B*⋯N4	0.96	2.85	3.126 (6)	98
C32—H32*A*⋯F6^iii^	0.96	2.40	3.196 (14)	140
C32—H32*A*⋯F7*A* ^iii^	0.96	2.33	3.19 (3)	150
C32—H32*B*⋯F3^v^	0.96	2.55	3.145 (15)	121
C32—H32*B*⋯F3*A* ^v^	0.96	2.32	3.11 (4)	140

Symmetry codes: (i) 
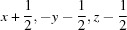
; (ii) 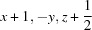; (iii) 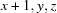; (iv) 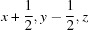; (v) 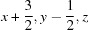.

**Table 2:** Experimental details

Crystal data	
Chemical formula	[Ni(C_30_H_35_N_5_)(C_2_H_3_N)](BF_4_)_2_
*M*_r_	739.01
Crystal system, space group	Monoclinic, *C* *c*
Temperature (K)	298
*a*, *b*, *c* (Å)	11.230 (3), 17.204 (5), 18.110 (6)
β (°)	103.248 (7)
*V* (Å^3^)	3405.7 (18)
*Z*	4
Radiation type	Mo *K*α
μ (mm^−1^)	0.65
Crystal size (mm)	0.30 × 0.20 × 0.10

Data collection	
Diffractometer	Bruker SMART CCD area detector
Absorption correction	Multi-scan (*SADABS*; Bruker, 2016 ▸)
*T*_min_, *T*_max_	0.847, 0.955
No. of measured, independent and observed [*I* > 2σ(*I*)] reflections	14710, 6476, 6304
*R*_int_	0.032
(sin θ/λ)_max_ (Å^−1^)	0.668

Refinement	
*R*[*F* ^2^ > 2σ(*F* ^2^)], *wR*(*F* ^2^), *S*	0.035, 0.092, 1.03
No. of reflections	6476
No. of parameters	520
No. of restraints	2
H-atom treatment	H-atom parameters constrained
Δρ_max_, Δρ_min_ (e Å^−3^)	0.29, −0.25
Absolute structure	Classical Flack (1983 ▸) method preferred over Parsons because s.u. lower.
Absolute structure parameter	−0.017 (12)

Computer programs: *SMART* and *SAINT* (Bruker, 2016 ▸), *SHELXTL* (Sheldrick, 2008 ▸) and *SHELXL2016* (Sheldrick, 2015 ▸).

## supplementary crystallographic information

### Crystal data

**Table:**

[Ni(C_30_H_35_N_5_)(C_2_H_3_N)](BF_4_)_2_	*F*(000) = 1528
*M**_r_* = 739.01	*D*_x_ = 1.441 Mg m^−^^3^
Monoclinic, *C**c*	Mo *K*α radiation, λ = 0.71073 Å
*a* = 11.230 (3) Å	Cell parameters from 8509 reflections
*b* = 17.204 (5) Å	θ = 2.3–28.4°
*c* = 18.110 (6) Å	µ = 0.65 mm^−^^1^
β = 103.248 (7)°	*T* = 298 K
*V* = 3405.7 (18) Å^3^	Block, blue
*Z* = 4	0.30 × 0.20 × 0.10 mm

### Data collection

**Table:**

Bruker SMART CCD area detector diffractometer	6304 reflections with *I* > 2σ(*I*)
Radiation source: sealed tube	*R*_int_ = 0.032
phi and ω scans	θ_max_ = 28.4°, θ_min_ = 3.5°
Absorption correction: multi-scan (SADABS; Bruker, 2016)	*h* = −14→11
*T*_min_ = 0.847, *T*_max_ = 0.955	*k* = −22→22
14710 measured reflections	*l* = −24→24
6476 independent reflections	

### Refinement

**Table:**

Refinement on *F*^2^	H-atom parameters constrained
Least-squares matrix: full	*w* = 1/[σ^2^(*F*_o_^2^) + (0.060*P*)^2^ + 1.7104*P*] where *P* = (*F*_o_^2^ + 2*F*_c_^2^)/3
*R*[*F*^2^ > 2σ(*F*^2^)] = 0.035	(Δ/σ)_max_ = 0.001
*wR*(*F*^2^) = 0.092	Δρ_max_ = 0.29 e Å^−^^3^
*S* = 1.03	Δρ_min_ = −0.25 e Å^−^^3^
6476 reflections	Extinction correction: SHELXL2016 (Sheldrick, 2015), Fc^*^=kFc[1+0.001xFc^2^λ^3^/sin(2θ)]^-1/4^
520 parameters	Extinction coefficient: 0.0091 (17)
2 restraints	Absolute structure: Classical Flack method preferred over Parsons because s.u. lower.
Hydrogen site location: mixed	Absolute structure parameter: −0.017 (12)

### Special details

**Table:**

Geometry. All esds (except the esd in the dihedral angle between two l.s. planes) are estimated using the full covariance matrix. The cell esds are taken into account individually in the estimation of esds in distances, angles and torsion angles; correlations between esds in cell parameters are only used when they are defined by crystal symmetry. An approximate (isotropic) treatment of cell esds is used for estimating esds involving l.s. planes.

### Fractional atomic coordinates and isotropic or equivalent isotropic displacement parameters (Å^2^)

**Table:**

	*x*	*y*	*z*	*U*_iso_*/*U*_eq_	Occ. (<1)
Ni1	0.73459 (2)	−0.19011 (2)	0.24468 (2)	0.02752 (12)	
N1	0.6542 (3)	−0.29110 (15)	0.18272 (14)	0.0333 (5)	
N2	0.5812 (3)	−0.20749 (17)	0.28899 (14)	0.0331 (5)	
N3	0.7742 (3)	−0.09858 (14)	0.33073 (13)	0.0325 (5)	
N4	0.8969 (3)	−0.17465 (19)	0.21169 (18)	0.0395 (6)	
N5	0.8531 (3)	−0.25827 (15)	0.33616 (14)	0.0361 (5)	
N6	0.6119 (2)	−0.11087 (13)	0.16863 (13)	0.0303 (5)	
C1	0.7455 (4)	−0.2798 (2)	0.0724 (2)	0.0515 (9)	
H1A	0.727964	−0.299078	0.021314	0.077*	
H1B	0.830687	−0.287160	0.095248	0.077*	
H1C	0.726121	−0.225425	0.072011	0.077*	
C2	0.6698 (3)	−0.32319 (17)	0.11726 (17)	0.0354 (6)	
C3	0.6181 (4)	−0.3937 (2)	0.0906 (2)	0.0460 (8)	
H3A	0.631892	−0.414716	0.045975	0.055*	
C4	0.5459 (4)	−0.4324 (2)	0.1306 (3)	0.0599 (10)	
H4A	0.510311	−0.479825	0.113295	0.072*	
C5	0.5270 (5)	−0.3999 (2)	0.1971 (3)	0.0569 (10)	
H5A	0.478334	−0.425011	0.224961	0.068*	
C6	0.5816 (3)	−0.32968 (18)	0.22115 (19)	0.0382 (6)	
C7	0.5658 (4)	−0.29254 (19)	0.29387 (19)	0.0421 (7)	
H7A	0.485006	−0.304199	0.301634	0.051*	
H7B	0.626011	−0.313171	0.336524	0.051*	
C8	0.6011 (3)	−0.1684 (2)	0.36392 (17)	0.0405 (7)	
H8A	0.653812	−0.200003	0.402342	0.049*	
H8B	0.523544	−0.161637	0.378152	0.049*	
C9	0.6601 (3)	−0.08985 (19)	0.35846 (17)	0.0379 (6)	
H9A	0.603190	−0.056630	0.324016	0.046*	
H9B	0.678916	−0.065285	0.407993	0.046*	
C10	0.8122 (4)	−0.02283 (16)	0.30210 (16)	0.0376 (6)	
H10A	0.747853	−0.006513	0.259492	0.045*	
H10B	0.884650	−0.032304	0.282792	0.045*	
C11	0.8397 (3)	0.04463 (17)	0.35776 (17)	0.0387 (6)	
C12	0.9533 (4)	0.0523 (2)	0.4080 (2)	0.0506 (8)	
H12A	1.011855	0.013546	0.410691	0.061*	
C13	0.9795 (5)	0.1177 (3)	0.4540 (2)	0.0639 (12)	
H13A	1.055817	0.122176	0.487139	0.077*	
C14	0.8960 (6)	0.1749 (3)	0.4515 (3)	0.0665 (15)	
H14A	0.915001	0.218324	0.482537	0.080*	
C15	0.7850 (7)	0.1685 (3)	0.4038 (4)	0.0741 (17)	
H15A	0.727325	0.207599	0.402322	0.089*	
C16	0.7555 (5)	0.1034 (2)	0.3562 (3)	0.0588 (10)	
H16A	0.678691	0.099906	0.323433	0.071*	
C17	0.8769 (5)	−0.3819 (2)	0.2717 (2)	0.0618 (11)	
H17A	0.947332	−0.415222	0.279414	0.093*	
H17B	0.877006	−0.347513	0.229995	0.093*	
H17C	0.803989	−0.412999	0.260617	0.093*	
C18	0.8809 (4)	−0.3350 (2)	0.3424 (2)	0.0462 (7)	
C19	0.9179 (5)	−0.3706 (2)	0.4121 (3)	0.0648 (12)	
H19A	0.935934	−0.423435	0.414915	0.078*	
C20	0.9283 (6)	−0.3275 (3)	0.4778 (3)	0.0703 (14)	
H20A	0.946599	−0.351625	0.525012	0.084*	
C21	0.9112 (4)	−0.2481 (2)	0.4721 (2)	0.0530 (9)	
H21A	0.923136	−0.217195	0.515292	0.064*	
C22	0.8758 (3)	−0.21540 (18)	0.40064 (17)	0.0374 (6)	
C23	0.8742 (3)	−0.12851 (18)	0.39211 (17)	0.0391 (6)	
H23A	0.866918	−0.105272	0.439683	0.047*	
H23B	0.951744	−0.111991	0.382379	0.047*	
C24	0.4730 (3)	−0.1755 (2)	0.23570 (19)	0.0376 (6)	
H24A	0.416027	−0.156355	0.264446	0.045*	
H24B	0.432739	−0.217051	0.203176	0.045*	
C25	0.5017 (3)	−0.11045 (16)	0.18656 (15)	0.0328 (5)	
C26	0.4120 (4)	−0.0560 (2)	0.1587 (2)	0.0485 (8)	
H26A	0.338511	−0.056083	0.174092	0.058*	
C27	0.4340 (4)	−0.0012 (2)	0.1072 (3)	0.0581 (10)	
H27A	0.374968	0.035766	0.087176	0.070*	
C28	0.5429 (4)	−0.00225 (18)	0.0866 (2)	0.0481 (8)	
H28A	0.557883	0.033626	0.051338	0.058*	
C29	0.6327 (3)	−0.05676 (16)	0.11773 (16)	0.0356 (6)	
C30	0.7518 (4)	−0.0552 (2)	0.0947 (2)	0.0522 (9)	
H30A	0.749443	−0.015746	0.056818	0.078*	
H30B	0.766266	−0.104850	0.074227	0.078*	
H30C	0.816492	−0.043836	0.138065	0.078*	
C31	0.9942 (4)	−0.1705 (3)	0.2068 (2)	0.0524 (9)	
C32	1.1190 (6)	−0.1620 (6)	0.1991 (5)	0.116 (3)	
H32A	1.151132	−0.112648	0.219080	0.139*	
H32B	1.121102	−0.165218	0.146450	0.139*	
H32C	1.167603	−0.203008	0.226670	0.139*	
B1	−0.2984 (7)	0.1828 (4)	0.0952 (4)	0.0668 (16)	
B2	0.2397 (7)	−0.0699 (4)	0.3807 (4)	0.0734 (15)	
F1	−0.2730 (12)	0.1428 (8)	0.1633 (4)	0.118 (4)	0.71 (2)
F2	−0.4166 (9)	0.1922 (5)	0.0757 (8)	0.124 (5)	0.71 (2)
F3	−0.2264 (10)	0.2458 (7)	0.1037 (7)	0.111 (4)	0.71 (2)
F4	−0.275 (2)	0.1337 (9)	0.0395 (8)	0.093 (5)	0.71 (2)
F1A	−0.233 (2)	0.181 (2)	0.1638 (17)	0.138 (11)	0.29 (2)
F2A	−0.411 (3)	0.185 (2)	0.111 (3)	0.21 (2)	0.29 (2)
F3A	−0.287 (4)	0.2622 (10)	0.067 (2)	0.127 (12)	0.29 (2)
F4A	−0.251 (6)	0.1297 (18)	0.054 (2)	0.098 (10)	0.29 (2)
F5	0.205 (2)	−0.1435 (7)	0.3810 (10)	0.208 (10)	0.611 (18)
F6	0.1540 (9)	−0.0300 (7)	0.3264 (5)	0.122 (4)	0.611 (18)
F7	0.3403 (11)	−0.0688 (7)	0.3563 (10)	0.135 (5)	0.611 (18)
F8	0.2472 (17)	−0.0380 (11)	0.4477 (6)	0.137 (6)	0.611 (18)
F5A	0.348 (2)	−0.105 (3)	0.414 (4)	0.30 (2)	0.389 (18)
F6A	0.1583 (13)	−0.1213 (13)	0.3817 (13)	0.125 (8)	0.389 (18)
F7A	0.246 (7)	−0.0368 (13)	0.3222 (13)	0.30 (3)	0.389 (18)
F8A	0.2379 (16)	−0.0186 (17)	0.4387 (13)	0.126 (8)	0.389 (18)

### Atomic displacement parameters (Å^2^)

**Table:**

	*U*^11^	*U*^22^	*U*^33^	*U*^12^	*U*^13^	*U*^23^
Ni1	0.02251 (17)	0.03240 (16)	0.02857 (16)	−0.00093 (15)	0.00773 (11)	0.00228 (14)
N1	0.0299 (13)	0.0365 (11)	0.0343 (11)	−0.0040 (11)	0.0090 (10)	0.0032 (9)
N2	0.0284 (14)	0.0418 (11)	0.0315 (11)	−0.0022 (12)	0.0119 (10)	0.0037 (10)
N3	0.0326 (14)	0.0335 (10)	0.0312 (10)	0.0008 (10)	0.0068 (10)	0.0013 (9)
N4	0.0288 (15)	0.0488 (13)	0.0434 (14)	−0.0049 (13)	0.0132 (12)	−0.0014 (12)
N5	0.0320 (13)	0.0372 (12)	0.0362 (11)	0.0008 (11)	0.0021 (10)	0.0009 (10)
N6	0.0281 (12)	0.0332 (10)	0.0294 (10)	−0.0016 (9)	0.0065 (9)	0.0017 (8)
C1	0.066 (3)	0.0537 (18)	0.0417 (15)	−0.0160 (18)	0.0256 (17)	−0.0068 (14)
C2	0.0337 (16)	0.0370 (12)	0.0347 (13)	−0.0021 (12)	0.0060 (12)	0.0009 (11)
C3	0.044 (2)	0.0420 (15)	0.0517 (18)	−0.0067 (15)	0.0094 (16)	−0.0100 (14)
C4	0.056 (3)	0.0379 (16)	0.088 (3)	−0.0169 (17)	0.022 (2)	−0.0129 (17)
C5	0.059 (3)	0.0411 (16)	0.078 (3)	−0.0205 (17)	0.031 (2)	−0.0019 (17)
C6	0.0331 (16)	0.0349 (13)	0.0491 (16)	−0.0040 (12)	0.0147 (13)	0.0065 (12)
C7	0.0426 (19)	0.0430 (14)	0.0469 (16)	−0.0064 (14)	0.0229 (15)	0.0086 (13)
C8	0.0397 (17)	0.0529 (16)	0.0331 (13)	−0.0016 (15)	0.0171 (13)	0.0012 (12)
C9	0.0375 (16)	0.0451 (14)	0.0323 (12)	0.0035 (13)	0.0104 (12)	−0.0047 (11)
C10	0.0492 (19)	0.0302 (12)	0.0334 (12)	−0.0008 (12)	0.0092 (12)	0.0013 (10)
C11	0.0458 (19)	0.0350 (12)	0.0376 (13)	−0.0027 (13)	0.0143 (13)	−0.0011 (11)
C12	0.055 (2)	0.0464 (17)	0.0496 (17)	−0.0074 (17)	0.0103 (17)	−0.0017 (14)
C13	0.076 (3)	0.069 (2)	0.0478 (18)	−0.029 (2)	0.016 (2)	−0.0131 (17)
C14	0.093 (4)	0.057 (2)	0.060 (2)	−0.020 (2)	0.038 (3)	−0.0246 (19)
C15	0.088 (5)	0.053 (2)	0.091 (4)	0.008 (3)	0.041 (4)	−0.021 (3)
C16	0.059 (3)	0.0444 (17)	0.074 (2)	0.0061 (18)	0.016 (2)	−0.0109 (17)
C17	0.067 (3)	0.0478 (19)	0.062 (2)	0.025 (2)	−0.003 (2)	−0.0082 (16)
C18	0.0418 (19)	0.0396 (14)	0.0519 (18)	0.0044 (15)	−0.0004 (15)	−0.0004 (14)
C19	0.077 (3)	0.0410 (18)	0.065 (2)	0.011 (2)	−0.006 (2)	0.0104 (17)
C20	0.094 (4)	0.057 (2)	0.048 (2)	0.011 (3)	−0.008 (2)	0.0191 (17)
C21	0.062 (3)	0.0508 (18)	0.0377 (15)	0.0027 (18)	−0.0049 (16)	0.0066 (13)
C22	0.0342 (16)	0.0367 (13)	0.0366 (13)	0.0000 (13)	−0.0017 (12)	0.0033 (11)
C23	0.0385 (17)	0.0373 (13)	0.0358 (13)	−0.0018 (13)	−0.0031 (12)	−0.0006 (11)
C24	0.0218 (14)	0.0504 (15)	0.0420 (15)	−0.0030 (12)	0.0104 (12)	0.0007 (12)
C25	0.0257 (14)	0.0377 (13)	0.0330 (12)	0.0018 (11)	0.0025 (10)	−0.0031 (10)
C26	0.0308 (17)	0.0517 (17)	0.0593 (19)	0.0097 (15)	0.0027 (15)	0.0021 (15)
C27	0.048 (2)	0.0453 (17)	0.071 (2)	0.0153 (17)	−0.0058 (18)	0.0078 (16)
C28	0.060 (2)	0.0326 (13)	0.0449 (16)	0.0005 (15)	−0.0022 (16)	0.0093 (12)
C29	0.0428 (18)	0.0313 (12)	0.0300 (11)	−0.0028 (12)	0.0030 (12)	0.0020 (10)
C30	0.055 (2)	0.0528 (18)	0.0553 (19)	−0.0033 (17)	0.0252 (18)	0.0177 (16)
C31	0.038 (2)	0.066 (2)	0.059 (2)	−0.0107 (18)	0.0219 (17)	−0.0207 (18)
C32	0.047 (3)	0.176 (7)	0.139 (6)	−0.035 (4)	0.050 (4)	−0.083 (6)
B1	0.062 (4)	0.071 (3)	0.062 (3)	−0.015 (3)	0.004 (3)	−0.014 (2)
B2	0.081 (4)	0.073 (3)	0.072 (3)	0.004 (3)	0.029 (3)	0.004 (3)
F1	0.127 (8)	0.174 (9)	0.049 (3)	0.013 (6)	0.014 (3)	0.000 (4)
F2	0.066 (5)	0.090 (5)	0.181 (10)	0.003 (4)	−0.044 (6)	−0.009 (5)
F3	0.106 (6)	0.105 (6)	0.136 (7)	−0.057 (5)	0.055 (5)	−0.073 (6)
F4	0.157 (13)	0.077 (5)	0.052 (3)	−0.032 (6)	0.036 (6)	−0.014 (3)
F1A	0.069 (12)	0.17 (3)	0.15 (2)	0.006 (12)	−0.033 (12)	−0.037 (17)
F2A	0.14 (2)	0.20 (3)	0.34 (5)	−0.10 (2)	0.18 (3)	−0.16 (3)
F3A	0.20 (3)	0.060 (6)	0.17 (2)	0.013 (11)	0.14 (2)	0.025 (10)
F4A	0.135 (19)	0.047 (8)	0.11 (2)	−0.006 (9)	0.017 (18)	−0.020 (10)
F5	0.35 (3)	0.080 (6)	0.159 (11)	−0.074 (10)	−0.018 (14)	0.039 (6)
F6	0.125 (7)	0.170 (9)	0.071 (4)	0.085 (7)	0.020 (4)	0.029 (4)
F7	0.098 (7)	0.134 (8)	0.207 (13)	0.026 (5)	0.105 (9)	−0.001 (7)
F8	0.174 (13)	0.183 (13)	0.053 (4)	−0.015 (9)	0.025 (5)	0.006 (6)
F5A	0.087 (14)	0.37 (5)	0.44 (6)	0.09 (2)	0.06 (3)	−0.03 (5)
F6A	0.056 (6)	0.179 (19)	0.151 (13)	−0.046 (8)	0.047 (8)	−0.033 (12)
F7A	0.73 (8)	0.102 (13)	0.113 (13)	−0.14 (3)	0.18 (3)	−0.021 (10)
F8A	0.061 (7)	0.163 (15)	0.155 (17)	−0.008 (8)	0.029 (9)	−0.090 (13)

### Geometric parameters (Å, º)

**Table:**

Ni1—N4	2.061 (3)	C15—H15A	0.9300
Ni1—N2	2.082 (3)	C16—H16A	0.9300
Ni1—N1	2.151 (3)	C17—C18	1.505 (5)
Ni1—N6	2.187 (3)	C17—H17A	0.9600
Ni1—N3	2.188 (2)	C17—H17B	0.9600
Ni1—N5	2.209 (3)	C17—H17C	0.9600
N1—C2	1.355 (4)	C18—C19	1.378 (6)
N1—C6	1.361 (4)	C19—C20	1.385 (7)
N2—C24	1.475 (5)	C19—H19A	0.9300
N2—C7	1.479 (4)	C20—C21	1.381 (6)
N2—C8	1.485 (4)	C20—H20A	0.9300
N3—C23	1.480 (4)	C21—C22	1.382 (4)
N3—C9	1.488 (4)	C21—H21A	0.9300
N3—C10	1.500 (4)	C22—C23	1.503 (4)
N4—C31	1.119 (5)	C23—H23A	0.9700
N5—C22	1.355 (4)	C23—H23B	0.9700
N5—C18	1.356 (4)	C24—C25	1.509 (4)
N6—C25	1.350 (4)	C24—H24A	0.9700
N6—C29	1.367 (3)	C24—H24B	0.9700
C1—C2	1.502 (4)	C25—C26	1.384 (5)
C1—H1A	0.9600	C26—C27	1.386 (6)
C1—H1B	0.9600	C26—H26A	0.9300
C1—H1C	0.9600	C27—C28	1.360 (7)
C2—C3	1.383 (4)	C27—H27A	0.9300
C3—C4	1.377 (5)	C28—C29	1.398 (5)
C3—H3A	0.9300	C28—H28A	0.9300
C4—C5	1.388 (6)	C29—C30	1.490 (5)
C4—H4A	0.9300	C30—H30A	0.9600
C5—C6	1.380 (5)	C30—H30B	0.9600
C5—H5A	0.9300	C30—H30C	0.9600
C6—C7	1.510 (4)	C31—C32	1.448 (6)
C7—H7A	0.9700	C32—H32A	0.9600
C7—H7B	0.9700	C32—H32B	0.9600
C8—C9	1.519 (5)	C32—H32C	0.9598
C8—H8A	0.9700	B1—F1A	1.29 (3)
C8—H8B	0.9700	B1—F2	1.303 (12)
C9—H9A	0.9700	B1—F3	1.339 (10)
C9—H9B	0.9700	B1—F4A	1.36 (4)
C10—C11	1.522 (4)	B1—F2A	1.36 (2)
C10—H10A	0.9700	B1—F1	1.384 (11)
C10—H10B	0.9700	B1—F4	1.385 (14)
C11—C16	1.380 (5)	B1—F3A	1.470 (16)
C11—C12	1.392 (6)	B2—F7A	1.219 (16)
C12—C13	1.391 (5)	B2—F6A	1.276 (17)
C12—H12A	0.9300	B2—F7	1.305 (10)
C13—C14	1.352 (8)	B2—F8	1.317 (14)
C13—H13A	0.9300	B2—F5	1.324 (12)
C14—C15	1.349 (9)	B2—F5A	1.36 (3)
C14—H14A	0.9300	B2—F8A	1.38 (2)
C15—C16	1.405 (7)	B2—F6	1.389 (10)
			
N4—Ni1—N2	174.24 (13)	C13—C14—H14A	120.2
N4—Ni1—N1	104.24 (12)	C14—C15—C16	120.7 (5)
N2—Ni1—N1	78.45 (10)	C14—C15—H15A	119.6
N4—Ni1—N6	102.02 (11)	C16—C15—H15A	119.6
N2—Ni1—N6	82.81 (10)	C11—C16—C15	120.5 (5)
N1—Ni1—N6	92.67 (10)	C11—C16—H16A	119.8
N4—Ni1—N3	93.70 (11)	C15—C16—H16A	119.8
N2—Ni1—N3	83.11 (10)	C18—C17—H17A	109.5
N1—Ni1—N3	160.94 (9)	C18—C17—H17B	109.5
N6—Ni1—N3	89.77 (9)	H17A—C17—H17B	109.5
N4—Ni1—N5	82.24 (12)	C18—C17—H17C	109.5
N2—Ni1—N5	92.54 (11)	H17A—C17—H17C	109.5
N1—Ni1—N5	94.07 (11)	H17B—C17—H17C	109.5
N6—Ni1—N5	170.89 (9)	N5—C18—C19	121.6 (3)
N3—Ni1—N5	81.87 (10)	N5—C18—C17	119.3 (3)
C2—N1—C6	117.5 (3)	C19—C18—C17	119.0 (4)
C2—N1—Ni1	131.5 (2)	C18—C19—C20	119.8 (4)
C6—N1—Ni1	110.9 (2)	C18—C19—H19A	120.1
C24—N2—C7	108.6 (3)	C20—C19—H19A	120.1
C24—N2—C8	111.0 (3)	C21—C20—C19	118.8 (4)
C7—N2—C8	112.9 (2)	C21—C20—H20A	120.6
C24—N2—Ni1	108.95 (18)	C19—C20—H20A	120.6
C7—N2—Ni1	106.5 (2)	C20—C21—C22	118.6 (4)
C8—N2—Ni1	108.8 (2)	C20—C21—H21A	120.7
C23—N3—C9	110.2 (2)	C22—C21—H21A	120.7
C23—N3—C10	109.6 (3)	N5—C22—C21	122.8 (3)
C9—N3—C10	111.4 (2)	N5—C22—C23	117.2 (3)
C23—N3—Ni1	106.27 (18)	C21—C22—C23	119.7 (3)
C9—N3—Ni1	105.24 (19)	N3—C23—C22	114.4 (3)
C10—N3—Ni1	113.98 (16)	N3—C23—H23A	108.7
C31—N4—Ni1	167.3 (4)	C22—C23—H23A	108.7
C22—N5—C18	117.7 (3)	N3—C23—H23B	108.7
C22—N5—Ni1	108.6 (2)	C22—C23—H23B	108.7
C18—N5—Ni1	131.8 (2)	H23A—C23—H23B	107.6
C25—N6—C29	117.8 (3)	N2—C24—C25	114.0 (3)
C25—N6—Ni1	109.39 (18)	N2—C24—H24A	108.7
C29—N6—Ni1	131.9 (2)	C25—C24—H24A	108.7
C2—C1—H1A	109.5	N2—C24—H24B	108.7
C2—C1—H1B	109.5	C25—C24—H24B	108.7
H1A—C1—H1B	109.5	H24A—C24—H24B	107.6
C2—C1—H1C	109.5	N6—C25—C26	123.1 (3)
H1A—C1—H1C	109.5	N6—C25—C24	118.0 (3)
H1B—C1—H1C	109.5	C26—C25—C24	118.8 (3)
N1—C2—C3	122.3 (3)	C25—C26—C27	118.6 (4)
N1—C2—C1	118.2 (3)	C25—C26—H26A	120.7
C3—C2—C1	119.4 (3)	C27—C26—H26A	120.7
C4—C3—C2	119.4 (3)	C28—C27—C26	119.1 (3)
C4—C3—H3A	120.3	C28—C27—H27A	120.5
C2—C3—H3A	120.3	C26—C27—H27A	120.5
C3—C4—C5	119.2 (3)	C27—C28—C29	120.6 (3)
C3—C4—H4A	120.4	C27—C28—H28A	119.7
C5—C4—H4A	120.4	C29—C28—H28A	119.7
C6—C5—C4	118.8 (3)	N6—C29—C28	120.7 (3)
C6—C5—H5A	120.6	N6—C29—C30	120.3 (3)
C4—C5—H5A	120.6	C28—C29—C30	119.0 (3)
N1—C6—C5	122.8 (3)	C29—C30—H30A	109.5
N1—C6—C7	116.3 (3)	C29—C30—H30B	109.5
C5—C6—C7	120.9 (3)	H30A—C30—H30B	109.5
N2—C7—C6	109.1 (2)	C29—C30—H30C	109.5
N2—C7—H7A	109.9	H30A—C30—H30C	109.5
C6—C7—H7A	109.9	H30B—C30—H30C	109.5
N2—C7—H7B	109.9	N4—C31—C32	177.5 (7)
C6—C7—H7B	109.9	C31—C32—H32A	110.0
H7A—C7—H7B	108.3	C31—C32—H32B	109.6
N2—C8—C9	108.6 (2)	H32A—C32—H32B	109.5
N2—C8—H8A	110.0	C31—C32—H32C	108.8
C9—C8—H8A	110.0	H32A—C32—H32C	109.5
N2—C8—H8B	110.0	H32B—C32—H32C	109.5
C9—C8—H8B	110.0	F2—B1—F3	118.9 (9)
H8A—C8—H8B	108.3	F1A—B1—F4A	107 (3)
N3—C9—C8	110.8 (2)	F1A—B1—F2A	99 (2)
N3—C9—H9A	109.5	F4A—B1—F2A	129 (3)
C8—C9—H9A	109.5	F2—B1—F1	107.0 (9)
N3—C9—H9B	109.5	F3—B1—F1	107.7 (8)
C8—C9—H9B	109.5	F2—B1—F4	103.1 (13)
H9A—C9—H9B	108.1	F3—B1—F4	111.7 (10)
N3—C10—C11	117.7 (2)	F1—B1—F4	108.1 (9)
N3—C10—H10A	107.9	F1A—B1—F3A	106.1 (18)
C11—C10—H10A	107.9	F4A—B1—F3A	111.3 (18)
N3—C10—H10B	107.9	F2A—B1—F3A	102 (2)
C11—C10—H10B	107.9	F7A—B2—F6A	122 (3)
H10A—C10—H10B	107.2	F7—B2—F8	115.4 (12)
C16—C11—C12	117.7 (3)	F7—B2—F5	106.9 (13)
C16—C11—C10	120.7 (4)	F8—B2—F5	110.6 (12)
C12—C11—C10	121.4 (3)	F7A—B2—F5A	111 (3)
C13—C12—C11	120.3 (4)	F6A—B2—F5A	105 (2)
C13—C12—H12A	119.9	F7A—B2—F8A	112.3 (16)
C11—C12—H12A	119.9	F6A—B2—F8A	107.4 (13)
C14—C13—C12	121.2 (5)	F5A—B2—F8A	97 (2)
C14—C13—H13A	119.4	F7—B2—F6	105.5 (9)
C12—C13—H13A	119.4	F8—B2—F6	109.6 (10)
C15—C14—C13	119.6 (4)	F5—B2—F6	108.6 (12)
C15—C14—H14A	120.2		
			
C6—N1—C2—C3	−2.1 (5)	C22—N5—C18—C19	7.0 (6)
Ni1—N1—C2—C3	173.2 (3)	Ni1—N5—C18—C19	−155.3 (4)
C6—N1—C2—C1	177.5 (3)	C22—N5—C18—C17	−170.0 (4)
Ni1—N1—C2—C1	−7.2 (5)	Ni1—N5—C18—C17	27.7 (6)
N1—C2—C3—C4	1.4 (6)	N5—C18—C19—C20	−0.4 (8)
C1—C2—C3—C4	−178.2 (4)	C17—C18—C19—C20	176.6 (5)
C2—C3—C4—C5	−0.2 (7)	C18—C19—C20—C21	−5.4 (9)
C3—C4—C5—C6	−0.2 (7)	C19—C20—C21—C22	4.5 (8)
C2—N1—C6—C5	1.7 (5)	C18—N5—C22—C21	−8.1 (6)
Ni1—N1—C6—C5	−174.5 (3)	Ni1—N5—C22—C21	158.1 (3)
C2—N1—C6—C7	−179.9 (3)	C18—N5—C22—C23	165.5 (3)
Ni1—N1—C6—C7	3.8 (4)	Ni1—N5—C22—C23	−28.3 (4)
C4—C5—C6—N1	−0.6 (7)	C20—C21—C22—N5	2.3 (7)
C4—C5—C6—C7	−178.9 (4)	C20—C21—C22—C23	−171.1 (4)
C24—N2—C7—C6	72.3 (3)	C9—N3—C23—C22	84.2 (3)
C8—N2—C7—C6	−164.3 (3)	C10—N3—C23—C22	−152.9 (3)
Ni1—N2—C7—C6	−44.9 (3)	Ni1—N3—C23—C22	−29.3 (3)
N1—C6—C7—N2	27.5 (4)	N5—C22—C23—N3	41.5 (4)
C5—C6—C7—N2	−154.1 (4)	C21—C22—C23—N3	−144.7 (4)
C24—N2—C8—C9	−78.0 (3)	C7—N2—C24—C25	−142.1 (3)
C7—N2—C8—C9	159.8 (3)	C8—N2—C24—C25	93.4 (3)
Ni1—N2—C8—C9	41.8 (3)	Ni1—N2—C24—C25	−26.5 (3)
C23—N3—C9—C8	−75.9 (3)	C29—N6—C25—C26	−3.1 (4)
C10—N3—C9—C8	162.3 (3)	Ni1—N6—C25—C26	167.6 (3)
Ni1—N3—C9—C8	38.3 (3)	C29—N6—C25—C24	173.2 (3)
N2—C8—C9—N3	−55.1 (4)	Ni1—N6—C25—C24	−16.2 (3)
C23—N3—C10—C11	−62.0 (4)	N2—C24—C25—N6	29.8 (4)
C9—N3—C10—C11	60.2 (4)	N2—C24—C25—C26	−153.7 (3)
Ni1—N3—C10—C11	179.1 (3)	N6—C25—C26—C27	2.9 (5)
N3—C10—C11—C16	−102.0 (4)	C24—C25—C26—C27	−173.3 (3)
N3—C10—C11—C12	82.8 (4)	C25—C26—C27—C28	−0.7 (6)
C16—C11—C12—C13	−0.5 (5)	C26—C27—C28—C29	−1.3 (6)
C10—C11—C12—C13	174.8 (3)	C25—N6—C29—C28	1.0 (4)
C11—C12—C13—C14	0.3 (6)	Ni1—N6—C29—C28	−167.1 (2)
C12—C13—C14—C15	0.2 (7)	C25—N6—C29—C30	−179.0 (3)
C13—C14—C15—C16	−0.5 (8)	Ni1—N6—C29—C30	13.0 (4)
C12—C11—C16—C15	0.2 (6)	C27—C28—C29—N6	1.1 (5)
C10—C11—C16—C15	−175.1 (4)	C27—C28—C29—C30	−178.9 (4)
C14—C15—C16—C11	0.2 (8)		

### Hydrogen-bond geometry (Å, º)

**Table:**

*D*—H···*A*	*D*—H	H···*A*	*D*···*A*	*D*—H···*A*
C1—H1*C*···N4	0.96	2.94	3.251 (6)	100
C3—H3*A*···F8^i^	0.93	2.57	3.457 (14)	159
C8—H8*B*···F7	0.97	2.57	3.371 (13)	140
C8—H8*B*···F5*A*	0.97	2.42	3.36 (4)	163
C9—H9*B*···F4^ii^	0.97	2.61	3.291 (16)	128
C10—H10*A*···N6	0.97	2.67	3.273 (5)	1213
C12—H12*A*···F8*A*^iii^	0.93	2.53	3.35 (2)	146
C17—H17*B*···N1	0.96	2.64	3.071 (7)	108
C21—H21*A*···F3^ii^	0.93	2.62	3.122 (10)	114
C23—H23*B*···F6*A*^iii^	0.97	2.33	3.241 (16)	157
C24—H24*A*···F7	0.97	2.53	3.443 (15)	156
C24—H24*B*···F3^iv^	0.97	2.32	3.179 (13)	148
C24—H24*B*···F1*A*^iv^	0.97	2.54	3.43 (3)	153
C28—H28*A*···F4^iii^	0.93	2.59	3.345 (19)	138
C30—H30*A*···F4^iii^	0.96	2.60	3.390 (17)	140
C30—H30*A*···F4*A*^iii^	0.96	2.50	3.26 (3)	136
C30—H30*B*···N4	0.96	2.85	3.126 (6)	98
C32—H32*A*···F6^iii^	0.96	2.40	3.196 (14)	140
C32—H32*A*···F7*A*^iii^	0.96	2.33	3.19 (3)	150
C32—H32*B*···F3^v^	0.96	2.55	3.145 (15)	121
C32—H32*B*···F3*A*^v^	0.96	2.32	3.11 (4)	140

Symmetry codes: (i) *x*+1/2, −*y*−1/2, *z*−1/2; (ii) *x*+1, −*y*, *z*+1/2; (iii) *x*+1, *y*, *z*; (iv) *x*+1/2, *y*−1/2, *z*; (v) *x*+3/2, *y*−1/2, *z*.
